# Factors influencing the length of stay in the psychiatric unit of a Ghanaian teaching hospital: a retrospective study

**DOI:** 10.1007/s00127-025-02889-1

**Published:** 2025-04-07

**Authors:** Stephen Wemakor, Kwabena Kusi-Mensah, John-Paul Omuojine, Richard Mensah, Ruth Owusu-Antwi

**Affiliations:** 1 Department of Psychiatry, Yale University, New Haven, Connecticut, United States of America; 2 Psychiatry Unit, Komfo Anokye Teaching Hospital, Kumasi, Ghana; 3 Department of Behavioural Sciences, School of Medicine and Dentistry, Kwame Nkrumah University of Science and Technology, Kumasi, Ghana; 4 Department of Psychiatry, University of Cambridge, Clifford Allbutt Building Cambridge Biomedical Campus CB2 OAH, Cambridge, UK

**Keywords:** Sub-Saharan Africa, Length of stay, Persons with psychiatric disorders, Hospitals, teaching, Mental disorders

## Abstract

**Purpose:**

The psychiatric length of stay (LOS) in community-based hospital facilities in sub-Saharan Africa reflects the quality of service delivery and the presence of resource challenges. This study aimed to determine the average LOS and identify factors associated with prolonged LOS in the psychiatric unit of a Ghanaian teaching hospital.

**Methods:**

The study analysed 1143 hospital discharge records of psychiatric inpatients at Komfo Anokye Teaching Hospital Psychiatric Unit from January 2016 to October 2020. LOS greater than the median of 10 days was classified as prolonged. We performed multivariable logistic regression to determine factors associated with prolonged LOS.

**Results:**

The mean LOS was 12 days, and the median LOS was 10 days. Bipolar and related disorders (aOR = 1.68 95% CI (1.28–2.21)), substance use disorders (aOR = 1.98 95% CI (1.19–3.30)), co-occurring mental health and substance use disorders (aOR = 2.30 95% CI (1.20–4.56)), and being discharged home directly (aOR = 1.91 95% CI (1.03–3.69)) was associated with a longer hospital stay, while suicide-related behaviour (aOR = 0.27 95% CI (0.09–0.72)) was associated with decreased odds of prolonged hospital stay.

**Conclusion:**

Possible interventions to reduce the length of psychiatric stay in the general hospital setting include improving functional integration of mental health into primary care and implementing transitional treatment programmes like partial hospitalisation and intensive outpatient treatment programmes. Improving access to residential substance use treatment is another intervention that can help decrease the burden of prolonged psychiatric stays.

**Supplementary Information:**

The online version contains supplementary material available at 10.1007/s00127-025-02889-1.

## Introduction

The number of mental health facilities in Africa is inadequate for the needs of the population [1]. The continent has one of the lowest median numbers of mental health facility beds at just 3.7 beds per 100,000 population [2]. To illustrate with specific examples, Ghana, with a population of over 30 million people, has just three mental hospitals [3]. With over 200 million people, Nigeria has just eight mental hospitals [4]. Zambia has just one mental hospital for nearly 18 million people [5]. In light of this shortage of mental hospitals, the promotion of deinstitutionalisation and the emergence of a balanced model of community-based mental healthcare in the African setting is a welcome one. The balanced care model of mental health provision is a community-based service close to the population served while minimising hospital stays to the briefest possible [6]. It involves the integration of mental health services into primary care and also advocates for the provision of inpatient psychiatric services in the general hospital setting. This has the potential to help address the problem of accessibility, which is one of the shortcomings of the asylum model that relies primarily on mental hospitals.

The shift towards community-based treatment means that settings such as psychiatric wards in general hospitals have become critical in the provision of ‘acute’ psychiatric care. However, they typically have fewer beds than mental hospitals [2]. Whether community-based mental healthcare will fulfil its promise remains to be seen. Currently, the continent has the lowest median number of inpatient mental health facilities (0.05 per 100,000 people) and beds (2.6 per 100,000 people) globally [2]. The lack of mental health infrastructure in Africa, coupled with the push towards community-based treatment, means the length of stay (LOS) in community-based hospital settings has assumed great significance. LOS can serve as a proxy for service efficiency and aid in the planning and distribution of hospital resources [7]. Information about factors associated with length of stay will help improve care and reduce healthcare system costs [8], primarily through shorter hospitalisation [9]. At the patient level, longer stays impose financial burdens, cause isolation from social contacts and may initiate maladaptive behaviour patterns [7].

Previous studies exploring LOS in the African setting have employed varying methodologies, and results demonstrated significant variation in the reporting of length of stay within and across clinical and geographical settings [8, 10–14]. Further, these studies showed heterogeneity in the factors associated with prolonged length of stay [8, 10–14]. To our knowledge, most studies examining factors related to psychiatric length of stay (LOS) in West Africa come from Nigeria, highlighting the need for data and insights from other countries in the region. The reasons mentioned above make results hard to generalise and highlight the need to investigate the factors influencing psychiatric inpatient stays in Ghana.

This study thus aimed to determine the average LOS in the psychiatric inpatient unit of a Ghanaian teaching hospital and identify factors associated with prolonged LOS. Here, we show the association between LOS and uncommonly assessed variables like the presence of suicide-related behaviours and discharge disposition. We also make the case for transitional treatment and residential programmes to help reduce LOS for specific diagnoses like substance use disorders. The study results will provide vital data for clinicians and mental health facility administrators in similar resource-challenged settings to improve the quality of care delivered and aid in planning mental health services.

## Methods

### Study design

This study was retrospective and cross-sectional, involving the analysis of discharge summary records of all individuals admitted to the Komfo Anokye Teaching Hospital Psychiatry Unit from 1 January 2016 to 30 October 2020. The data was collected between 2 November 2020 and 29 January 2021.

### Setting

The Komfo Anokye Teaching Hospital (KATH) is a prominent tertiary referral centre for Ghana’s northern and middle sectors. It serves 13 out of 16 regions (provinces) and even receives referrals from neighbouring countries. Its psychiatry unit was established in 1982 [15]. At the time of the study, the unit staff comprised a multidisciplinary team of psychiatrists, psychiatry residents, medical officers, psychologists, nurses, and occupational therapists. The unit has an 11-bed ward that can admit five males and six females and runs an open ward system. Patients accepted into the unit must have a caregiver present throughout their stay. The unit also provides 24-hour staffing at the hospital’s Accident and Emergency Centre and runs several clinics. In 2019, the unit began rolling out an electronic medical record system, but before this, all documentation had traditionally been done in paper charts and stored at various record sites [16]. At discharge, patient details, including demographic, clinical and discharge status information, are recorded in a discharge summary book.

### Participants

Records of all patients admitted to the psychiatry unit with documented admission and discharge dates were eligible for analysis. Each admission was considered a separate event, so readmissions were included in the study to capture outcomes that differed from those of the index admission. Patients with psychiatric comorbidities who were managed in non-psychiatric inpatient units of the hospital were not included in the study. 1143 records met the inclusion criteria.

### Variables

The study’s primary outcome variables were the mean length of stay and the proportion of the prolonged length of stay (LOS) in the psychiatric ward. The median LOS was used as a cut-off, and any LOS greater than the median was classified as “prolonged”. The median LOS was preferred over the mean LOS because the LOS data had a positively skewed distribution, suggesting that the former would be the more accurate measure of central tendency [17].

The exposure variables were demographic and clinical characteristics, including age, gender, employment status, diagnosis, presence of suicide-related behaviour, presence of co-occurring mental health and substance use disorders, history of previous admission and discharge disposition. The exposure variables were developed based on the review of existing literature and information obtained from the discharge summary record.

Age was categorised into three groups based on Centers for Medicare and Medicaid Services data groupings, as the data was non-normally distributed and had a positive skew [18]. The International Statistical Classification of Disease and Related Health Problems, 10th Revision (ICD 10) (World Health Organization, 2019) is the basis for diagnostic classification and coding in Ghana, and all diagnoses at the Psychiatry Unit are consistent with its criteria. For our analysis, these diagnoses were categorised into five groups: psychosis and related disorders (F20-F29), substance use disorders (F10-F19), depressive disorders (F32), bipolar and related disorders (F31), and other psychiatric disorders not captured in the prior groups. Discharge disposition was classified into six categories: discharged home, absconded, discharged against medical advice, transferred to a long-stay facility, transferred to the medical ward, and deceased. During multivariable logistic regression analysis, discharge disposition was categorised into “discharged home” and “other” because the other categories had low observation counts.

### Data sources/measurement

The discharge summary records of patients were the data source, and a co-author collated the data in Microsoft Excel [19]. The first author then cross-checked the data for completeness and accuracy.

### Statistical methods

Analyses were conducted using the R Statistical language software (version 4.3.2; R Core Team, 2023), *gtsummary*, and *report* packages [20–22]. The complete reproducible code with associated packages is available in the supplementary material as an appendix.

The variables were categorical and thus tabulated as frequencies. The chi-square test, Wilcoxon rank sum test, and Fisher’s exact test were used to test for bivariate associations between exposure variables and LOS.

Multivariable logistic regression was employed to estimate the association between exposure variables and the odds of prolonged LOS while adjusting for the potential confounding effects of other exposures. All exposure variables were included in the logistic regression analysis because they were deemed clinically significant. 0.93% of the data was missing, and given the low level of missingness in the dataset, we employed a listwise deletion approach to address the missing data during analysis. The exposure variables were evaluated for multicollinearity using the generalised variance inflation factor (GVIF). The likelihood ratio test demonstrated a chi-square test statistic of 132.2 with 12 degrees of freedom and a p-value of < 0.001. The logistic regression model was assessed to ensure it met other logistic regression model assumptions. Results are presented as odds ratios within 95% confidence intervals at a 95% significance level.

## Results

### Demographic and clinical characteristics

The mean age of the study sample was 32.6 years (standard deviation of 12.2 years), while the median age was 30 years (range 10–91 years). 53.0% of the study sample were female, and 55.0% of participants were employed. Psychosis and related disorders were the most common diagnoses with 544 (49.0%) cases, followed by bipolar and related disorders with 396 (35.0%) cases. Substance-related and depressive disorders accounted for 77 (6.9%) and 49 (4.4%) of cases, respectively. 30 cases (2.7%) exhibited suicide-related behaviour, while 45 (4.0%) patients had co-occurring mental health and substance use disorders. 1065 (95.0%) patients were discharged to their homes, while 20 (1.8%) patients were transferred to other non-psychiatric wards. 14 (1.3%) patients absconded, 11 (1.0%) patients were discharged against medical advice, and 8 (0.7%) were transferred to a long-stay psychiatric facility. During the period under review, two patients (0.2%) died while on admission. Tests of association revealed statistically significant associations between diagnosis (p-value < 0.001), presence of co-occurring mental health and substance use disorders (p-value 0.045), suicide-related behaviour (p-value 0.007), discharge disposition (p-value 0.004) and LOS. Table 1 shows the demographic and clinical characteristics of the study sample.

**Table 1:** Demographic and clinical characteristics

VARIABLE	*N*	OVERALL, *N* = 1,143^*1*^	LENGTH OF STAY		*P*-VALUE^2^
			**SHORT**, *N* = 602^*1*^	**LONG**, *N* = 541^*1*^	
**AGE GROUP (YEARS)**	1,137				0.310
<19		83 (7.3%)	40 (6.7%)	43 (8.0%)	
19–64		1,028 (90.0%)	542 (90.0%)	486 (90.0%)	
65+		26 (2.3%)	17 (2.8%)	9 (1.7%)	
*Data missing*		*6*	*3*	*3*	
**SEX**	1,143				0.490
Male		535 (47.0%)	276 (46.0%)	259 (48.0%)	
Female		608 (53.0%)	326 (54.0%)	282 (52.0%)	
**EMPLOYMENT STATUS**	1,129				0.820
Unemployed		511 (45.0%)	267 (45.0%)	244 (46.0%)	
Employed		618 (55.0%)	327 (55.0%)	291 (54.0%)	
*Data missing*		*14*	*8*	*6*	
**DIAGNOSIS**	1,118				< 0.001
Psychosis and Related Disorders		544 (49.0%)	309 (53.0%)	235 (44.0%)	
Bipolar and Related Disorders		396 (35.0%)	181 (31.0%)	215 (40.0%)	
Substance Use Disorders		77 (6.9%)	32 (5.5%)	45 (8.4%)	
Depressive Disorders		49 (4.4%)	27 (4.6%)	22 (4.1%)	
Other Psychiatric Disorders		52 (4.7%)	36 (6.2%)	16 (3.0%)	
*Data missing*		*25*	*17*	*8*	
**PREVIOUS ADMISSION**	1,143	153 (13.0%)	84 (14.0%)	69 (13.0%)	0.550
**CO-OCCURRING DISORDERS**	1,130	45 (4.0%)	17 (2.9%)	28 (5.2%)	0.045
*Data missing*		*13*	*10*	*3*	
**SUICIDE-RELATED BEHAVIOUR**	1,130	30 (2.7%)	23 (3.9%)	7 (1.3%)	0.007
*Data missing*		*13*	*10*	*3*	
**DISCHARGE DISPOSITION**	1,120				0.004
Discharged Home		1,065 (95.0%)	554 (94.0%)	511 (96.0%)	
Absconded		14 (1.3%)	7 (1.2%)	7 (1.3%)	
Discharged Against Medical Advice		11 (1.0%)	10 (1.7%)	1 (0.2%)	
Deceased		2 (0.2%)	1 (0.2%)	1 (0.2%)	
Transferred To Long Stay Psychiatric Facility		8 (0.7%)	2 (0.3%)	6 (1.1%)	
Transferred To Another Ward		20 (1.8%)	16 (2.7%)	4 (0.8%)	
*Data missing*		*23*	*12*	*11*	
**DISCHARGE DISPOSITION- combined**	1,120				0.052
*Other*		55 (4.9%)	36 (6.1%)	19 (3.6%)	
Discharge home		1,065 (95.0%)	554 (94.0%)	511 (96.0%)	
Data missing		*23*	*12*	*11*	

^*1*^ Frequency (%)

^*2*^ Pearson’s Chi-squared test; Wilcoxon rank sum test; Fisher’s exact test

### Length of stay

The mean length of stay was 12.2 days, with a standard deviation of 8.5 days. The median length of stay was 10 days, ranging from 1 to 62 days. 47.3% of stays were prolonged (i.e.> 10 days).

### Factors associated with length of stay

A multivariable logistic regression analysis was carried out, and all exposure variables were included. Adjusting for age, employment status, sex and history of previous admission, bipolar and related disorders (aOR = 1.68 95% CI (1.28–2.21)), substance use disorders (aOR = 1.98 95% CI (1.19–3.30)), co-occurring mental health and substance use disorders (aOR = 2.30 95% CI (1.20–4.56)), and discharge home compared to other dispositions (aOR = 1.91 95% CI (1.03–3.69)) significantly predicted odds of longer hospital stay, while suicide-related behaviour (aOR = 0.27 95% CI (0.09–0.72)) was associated with decreased odds of prolonged LOS. Table 2 shows the results of the multivariable logistic regression analysis of factors associated with prolonged length of stay.

**Table 2:** Multivariable logistic regression analysis for factors associated with prolonged length of stay

VARIABLE	OR^1^	95% CI^1^	*P*-VALUE
**AGE GROUP (YEARS)**			
< 19	—	—	
19–64	0.75	0.46, 1.22	0.300
65+	0.50	0.19, 1.28	0.200
**SEX**			
Male	—	—	
Female	1.02	0.79, 1.32	0.900
**EMPLOYMENT STATUS**			
Unemployed	—	—	
Employed	0.92	0.71, 1.19	0.500
**DIAGNOSIS**			
Psychotic Disorders	—	—	
Bipolar And Related Disorders	1.68	1.28, 2.21	< 0.001
Substance Use Disorders	1.98	1.19, 3.30	0.008
Depressive Disorders	1.68	0.84, 3.41	0.140
Other Psychiatric Disorders	0.63	0.32, 1.18	0.200
**PREVIOUS ADMISSION**			
No	—	—	
Yes	0.82	0.57, 1.17	0.300
**CO-OCCURRING DISORDERS**			
No	—	—	
Yes	2.30	1.20, 4.56	0.014
**SUICIDE-RELATED BEHAVIOUR**			
No	—	—	
Yes	0.27	0.09, 0.72	0.014
**DISCHARGE DISPOSITION**			
Other	—	—	
Discharged Home	1.91	1.03, 3.69	0.045

^*1*^ OR = Odds Ratio, CI = Confidence Interval

### Other analyses

The mean and median length of stay for the various diagnoses and other exposure variables can be found in Table 1 of the supplementary material.

## Discussion

This study aimed to identify the factors associated with prolonged LOS in the ward of the KATH Psychiatry Unit. We analysed the unit’s discharge summary records and found that the mean length of stay was 12.2 days (median of 10 days), and almost half (47.3%) of the admissions were prolonged. Patients with primary diagnoses of bipolar and related disorders and substance use disorder had increased odds of prolonged hospital stay compared to those with psychosis and related disorders. Patients with co-occurring mental health and substance use disorders also had increased odds of prolonged hospital stay. Moreover, patients who were discharged home had increased odds of having experienced a prolonged hospital stay, while suicide-related behaviour was associated with decreased odds of prolonged hospital stay.

The mean length of stay of 12 days is at the lower end of the range internationally, especially when compared to high-income countries. In the United States, for instance, the average psychiatric LOS in primarily general hospital settings ranges between 7 and 25 days [23]. In high-income European countries, the average LOS is 39.4 days [24]. Our study’s mean length of stay is also lower than that observed in studies of similar psychiatric inpatient settings in sub-Saharan Africa [11, 12, 14, 25, 26]. This finding could be related to the number of beds available in the unit. The KATH Psychiatry Unit has 11 beds, the lowest compared to the abovementioned studies. Bed availability has been demonstrated to have a positive relationship with length of stay [27]. For a minor psychiatric unit ward in a tertiary health facility that serves a large catchment area, including cross-border populations [28], admission demands are far more than the bed capacity. There is immense pressure to admit patients and ease the burden on upstream points like the Accident and Emergency Centre, where patients requiring admission are boarded until bed space is available at the ward. Such constraints and hospital-level expectations could influence practice styles to shorten the duration of hospital stay [29]. This finding highlights the need to increase the number of psychiatric beds to make inpatient care accessible to more patients without compromising the quality of care [30].

Our finding of bipolar and related disorders being associated with prolonged stay differs from the results of other studies, which typically found psychotic disorders to be most associated with increased LOS [10, 14, 23], and we theorise that this may be related to the history of previous admissions among this patient population. In a separate multivariable logistic regression analysis, we found that patients with bipolar and related disorders had 1.69 times the odds of having had a previous psychiatric admission compared to patients with psychotic disorders, and this was statistically significant (see Table 2 in the supplementary material). Some authors have reported an association between shorter lengths of stay and increased readmission rates [31, 32]. It is, therefore, possible that patients with bipolar disorders tend to be admitted longer because of their history of previous admissions. Additionally, some studies have found an association between a diagnosis of bipolar disorder and aggression, with Barlow et al.‘s study being of an inpatient population [33, 34]. Thus, admitting patients with bipolar disorder longer could also be a risk mitigation measure to keep the larger community and the patients themselves safe. Longer hospital stays would provide the needed time to stabilise patients, adjust medications, arrange for comprehensive follow-up care, and hopefully reduce the risk of future readmission.

Other studies have found substance use to be associated with decreased length of stay [35, 36]. Our study found the opposite, and this was expected given this particular study setting and its practice philosophy for managing substance use disorders. The protocol for treating patients with substance use disorders on the unit ward involves an initial period of inpatient medically supervised withdrawal, followed by weeks of inpatient substance use rehabilitation leading to prolonged hospital stay. This management strategy could also explain why patients with co-occurring mental health and substance use disorders have higher odds of protracted hospital stay. This treatment approach for substance use disorders is a local innovation developed as an alternative to “step down” care, which is not readily available. It also highlights the non-existence of transitional treatment programmes like substance abuse intensive outpatient programmes and partial hospitalisation programmes, as well as the relative scarcity of residential substance use treatment programmes, with the available ones typically out of financial and geographical reach for most patients. Establishing transitional treatment programmes will offer safe diversion alternatives and free up much-needed bed space. Additionally, transitional treatment approaches like intensive outpatient programmes are associated with additional benefits like decreased emergency department visits and hospitalisations and reduced clinic and hospital-level costs [37].

Our finding regarding suicide-related behaviour and shorter lengths of stay is consistent with that of other authors, although these are studies in settings outside Africa [38, 39]. The typical suicide-related behaviour resulting in admission to the unit is a suicide attempt, usually via deliberate self-poisoning attributable to a situational crisis. Survivors of violent suicide attempts, such as the use of firearms, which are more likely to be associated with a prolonged hospital stay, were typically managed as consult cases on the non-psychiatric wards where their physical injuries could be better addressed. Compared to patients admitted for conditions like psychosis and bipolar disorder, patients admitted to the unit for suicide-related behaviours generally did not need extensive medication titration or exhibit other factors that would contribute to prolonged hospital stays. Collaborating with such patients to address underlying causes and develop safety plans typically resulted in relatively early discharge, following which they were enrolled in the outpatient clinic for further management. We believe this finding is significant because studies examining length of stay in the sub-Saharan African setting have generally not reported on its association with suicide-related behaviour. This is not surprising because the lack of robust data concerning suicide on the continent is well documented [40]. Our finding also highlights the need for further local studies in the area of suicide and related behaviours, including culturally appropriate interventions that can mitigate risk and the effect of hospitalisation on further suicide-related behaviour.

95% of patients admitted to the unit were stabilised and discharged home in presumably stable condition. The stay for other discharge dispositions was shorter than for patients with a routine discharge home, which seems counterintuitive. However, a previous study has highlighted how patients with longer stays are more likely to be stable at discharge [41]. It is also important to note that the other disposition options signalled a truncation of admission in most cases, including abscondment, discharge against medical advice, and transfers from the ward. Absconding, for example, is associated with decreased duration of hospital stay [42]. A qualitative study from Uganda highlights motivations for absconding, including poor resources and services, mental illness symptoms, and experiences with caregivers [43]. Other studies mention boredom, confinement, frustration, and the need to complete tasks [44, 45]. These factors are possible reasons why patients would abscond from the ward and shorten LOS. Also, the open ward system run by the psychiatry unit makes escape easier for patients who are motivated to do so. Discharge against medical advice is also associated with a shorter duration of hospitalisation, and factors related to this phenomenon include the sense of improvement, employment-related issues, and lack of amenities [46]. These factors are consistent with the experiences in the unit. Also, belief systems, especially about the causation of psychiatric disorders, lead to requests for discharge from the unit to seek spiritual and alternative medicine solutions. Additionally, when faced with financial constraints and the prospect of loss of income, caregivers will advocate for discharge against medical advice. Finally, patients who are assessed as inappropriate for management on the unit because of factors like medical comorbidity or substance use disorder requiring a longer duration of treatment are transferred to the appropriate treatment setting, thus shortening the duration of stay on the unit.

### Limitations

This study was retrospective and relied on discharge records; inaccurate or incomplete documentation in the records has the potential to bias the study results. The study’s cross-sectional nature prevented the assessment of causality between the length of stay and the variables analysed. It also meant that there was no longitudinal follow-up to determine which factors consistently influenced LOS over time.

0.93% of the data was missing, and some variables, like discharge disposition, had low observation counts, which could reduce the statistical power of the analysis.

The discharge records we examined lacked comprehensiveness, which hindered our ability to analyse certain variables identified in other studies as having significant associations with length of stay (LOS) [47]. For instance, we did not have information on specific demographic variables such as religion, education, and marital status, which affected the face validity of our results. Moreover, we could not analyse the impact of clinical variables like the nature of admission (voluntary vs. involuntary), use of restraints, and extrapyramidal side effects that could have influenced the study results. Social and economic factors, such as family involvement and financial constraints, might have affected LOS but were not measured and, therefore, not analysed in this study. The diagnoses included in the discharge records were based on clinical assessments without standardised diagnostic tools, which could result in classification errors.

Our study was also limited to a single setting, which may restrict the generalizability of our findings to other settings, such as standalone psychiatric hospitals or rehabilitation centres. Nonetheless, we have reasonable confidence that the study setting is representative of psychiatric units in other general hospitals in Ghana, allowing the applicability of our study findings in that context.

### Implications and future directions

The study results highlight the need to increase bed space in community-based inpatient psychiatric units. They also emphasise the need to scale up alternative treatment settings like intensive outpatient programmes, partial hospitalisation programmes and residential substance use programmes to offer meaningful options for patients and decrease reliance on community-based inpatient beds.

Further research may explore more patient, institutional, and practitioner factors affecting LOS and clinical interventions to safely reduce the length of hospitalisation and strengthen community-based outpatient care systems.

## Conclusions

Our study provides insights into the factors influencing the duration of psychiatric inpatient stays in a resource-limited setting. With an average length of stay of 12.2 days and nearly half of the admissions being extended, our findings emphasise the impact of diagnosis, co-occurring disorders, and discharge disposition on the length of hospital stays. Notably, patients with bipolar and related disorders, substance use disorders, and co-occurring mental health and substance use disorders had higher odds of extended admissions, while suicide-related behaviour was associated with shorter stays.

The results underscore significant gaps in Ghana’s psychiatric care infrastructure, including the limited availability of psychiatric beds and the lack of transitional treatment programmes for substance use disorders. Expanding community-based treatment options, implementing intensive outpatient programmes, and increasing inpatient capacity could enhance service efficiency while preserving quality care. Furthermore, additional research into culturally relevant suicide interventions is required.

By tackling these challenges, policymakers and healthcare providers can improve the delivery of mental health services in Ghana and other resource-limited settings, ultimately enhancing patient outcomes and optimising hospital resources.

## Electronic supplementary material

Below is the link to the electronic supplementary material.

Supplementary Material 1

## Data Availability

The data supporting the findings in this study are openly available in figshare at https://figshare.com/ with doi: 10.6084/m9.figshare.24970725.
