# Supplementary material for: Factors influencing the length of stay in the psychiatric unit of a Ghanaian teaching hospital: a retrospective study
Source: Soc Psychiatry Psychiatr Epidemiol. 2025 Apr 7;60(9):2089–97. doi: 10.1007/s00127-025-02889-1 (PMC12378297; doi:10.1007/s00127-025-02889-1)
Supplement: Supplementary file 1 — Supplementary Material 1 [file 127_2025_2889_MOESM1_ESM.docx]

**SUPPLEMENTARY INFORMATION:**

**Determinants of prolonged stay in the psychiatric unit of a Ghanaian teaching hospital: A retrospective study.**

**Journal name: Social Psychiatry and Psychiatric Epidemiology**

**Author Information**

Stephen Wemakor [0000-0001-7087-6364]^1,2^, Kwabena Kusi-Mensah [0000-0003-0165-8405]*^3,4^, John-Paul Omuojine [0000-0001-7320-6104]^2,3^, Richard Mensah [0009-0002-1612-3227]^2^, Ruth Owusu-Antwi [0000-0001-9657-1956]^3^

^1^ Department of Psychiatry, Yale University, New Haven, Connecticut, United States of America.

^2^ Psychiatry Unit, Komfo Anokye Teaching Hospital, Kumasi, Ghana.

^3^ Department of Behavioural Sciences, School of Medicine and Dentistry, Kwame Nkrumah University of Science and Technology, Kumasi, Ghana.

^4^ Department of Psychiatry, University of Cambridge, Cambridge, United Kingdom.

^*^  Corresponding author

E-mail: [kpk27@cam.ac.uk](mailto:kpk27@cam.ac.uk)

**TABLE 1: Mean and median LOS disaggregated by patient variables.**

| **VARIABLE** | **MEAN LOS IN DAYS (SD)^1^** | **MEDIAN LOS IN DAYS (IQR)** **^1^** |
| --- | --- | --- |
| AGE GROUP (YEARS) |  |  |
| <19 | 13.4 (9.3) | 11.0 (6.0, 20.5) |
| 19-64 | 12.1 (8.3) | 10.0 (6.0, 16.0) |
| 65+ | 11.1 (10.6) | 7.0 (5.0, 13.0) |
| *Data Missing* | *6* | *6* |
| SEX |  |  |
| Male | 12.8 (8.9) | 10.0 (6.0, 17.0) |
| Female | 11.8 (8.0) | 10.0 (6.0, 16.0) |
| EMPLOYMENT STATUS |  |  |
| Unemployed | 12.5 (8.6) | 10.0 (6.0, 16.0) |
| Employed | 12.1 (8.4) | 10.0 (6.0, 16.0) |
| *Data Missing* | *14* | *14* |
| DIAGNOSIS |  |  |
| Psychotic and Related Disorders | 11.5 (7.8) | 9.0 (6.0, 15.0) |
| Other Psychiatric Disorders | 9.3 (7.7) | 7.0 (4.0, 12.0) |
| Bipolar And Related Disorders | 13.1 (8.6) | 11.0 (7.0, 17.0) |
| Depressive Disorders | 11.4 (9.3) | 8.0 (4.0, 16.0) |
| Substance Use Disorders | 16.0 (11.2) | 12.0 (7.0, 23.0) |
| *Data Missing* | *25* | *25* |
| CO-OCCURRING DISORDERS |  |  |
| No | 12.2 (8.5) | 10.0 (6.0, 16.0) |
| Yes | 14.2 (8.9) | 11.0 (7.0, 20.0) |
| *Data Missing* | *13* | *13* |
| PREVIOUS ADMISSION |  |  |
| No | 12.3 (8.6) | 10.0 (6.0, 16.0) |
| Yes | 11.7 (7.8) | 10.0 (7.0, 15.0) |
| SUICIDE-RELATED BEHAVIOR |  |  |
| No | 12.4 (8.5) | 10.0 (6.0, 16.0) |
| Yes | 8.2 (6.9) | 6.0 (3.0, 9.8) |
| *Data Missing* | *13* | *13* |
| DISCHARGE DISPOSITION |  |  |
| Discharged | 12.4 (8.5) | 10.0 (6.0, 16.0) |
| Absconded | 11.6 (9.6) | 8.5 (5.2, 14.2) |
| Discharged Against Medical Advice | 5.3 (4.0) | 5.0 (1.5, 9.0) |
| Deceased | 13.5 (16.3) | 13.5 (7.8, 19.2) |
| Transferred To Long Stay Psychiatric Facility | 17.6 (11.0) | 19.0 (10.0, 25.0) |
| Transferred To Medical Ward | 8.5 (5.0) | 7.0 (5.0, 10.0) |
| *Data Missing* | *23* | *23* |

*^1^* SD = Standard Deviation, IQR = Interquartile Range

**TABLE 2: Multivariable logistic regression analysis results for factors associated with history of previous admission.**

| **Variable** | **OR***1* | **95% CI** *^1^* | **p-value** |
| --- | --- | --- | --- |
| DIAGNOSIS |  |  |  |
| Psychotic Disorders | — | — |  |
| Bipolar And Related Disorders | 1.69 | 1.17, 2.47 | 0.006 |
| Depressive Disorders | 0.19 | 0.01, 1.01 | 0.12 |
| Other Psychiatric Disorders | 0.28 | 0.04, 0.97 | 0.090 |
| Substance Use Disorders | 1.55 | 0.76, 3.00 | 0.2 |
| EMPLOYMENT STATUS |  |  |  |
| Unemployed | — | — |  |
| Employed | 0.81 | 0.57, 1.17 | 0.3 |
| CO-OCCURRING DISORDERS |  |  |  |
| No | — | — |  |
| Yes | 0.46 | 0.11, 1.35 | 0.2 |
| AGE GROUP (YEARS) |  |  |  |
| <19 | — | — |  |
| 19-64 | 2.53 | 1.07, 7.46 | 0.055 |
| 65+ | 1.62 | 0.22, 8.38 | 0.6 |
| SEX |  |  |  |
| Female | — | — |  |
| Male | 0.82 | 0.57, 1.19 | 0.3 |
| DISCHARGE DISPOSITION |  |  |  |
| Other | — | — |  |
| Discharged Home | 0.51 | 0.25,1.14 | 0.082 |
| LENGTH OF STAY |  |  |  |
| Long | — | — |  |
| Short | 1.21 | 0.85, 1.73 | 0.3 |
| SUICIDE-RELATED BEHAVIOR |  |  |  |
| No | — | — |  |
| Yes | 0.56 | 0.03, 3.27 | 0.6 |

*^1^* OR = Odds Ratio, CI = Confidence Interval

**APPENDIX 1: R Codes used in statistical analysis with associated package references.**

##################

#days=LOS (continuous variable)

#dx=diagnosis

#ddx=co-occurring disorders

#readmission=previous admission

#remarks=discharge disposition

#suicide=suicide-related behaviour

#work=employment status (employed, unemployed)

#EMP=employment status (formal employment, nonformal employment, unemployed)

#clos=LOS (short, long)

#newdata=dataset/dataframe

####################

library(readxl)

newdata <- read_excel("Library/Mobile Documents/com~apple~CloudDocs/R RESEARCH

WORK/newdata.xlsx")

View(newdata)

########################################################

#selecting variables to be analyzed

newdata = subset(newdata, select = c(readmission, age, sex, EMP, days, clos, dx, ddx, suicide, remarks)) View(newdata)

#######################################################

#creating age group categories install.packages("dplyr")

library(dplyr)

newdata= newdata %>%

mutate(

# Create categories agegroup = dplyr::case_when(

age <= 18 ~ "<19", age > 18 & age <= 64 ~ "19-64",

age > 64 ~ "65+",

),

# Convert to factor agegroup = factor( agegroup,

level = c("<19", "19-64","65+")

))

#Create a new column "work" from existing ones using ifelse

newdata$work = ifelse(newdata$EMP == "UNEMPLOYED","UNEMPLOYED","EMPLOYED")

#Remember to convert #to factor

newdata$work = as.factor(newdata$work)

#Create a new column "discharge" from existing ones using ifelse

newdata$discharge = ifelse(newdata$remarks == "DISCHARGED","DISCHARGED","OTHER")

#Remember to convert #to factor

newdata$discharge = as.factor(newdata$discharge)

#attempting a new means of recategorizing vectors

#i dont think i need to load any packages..

newdata$dx <- ifelse(newdata$dx == "PSYCHOTIC", "PSYCHOTIC", ifelse(newdata$dx == "BIPOL_MOOD", "BIPOL_MOOD",

ifelse(newdata$dx == "DEPRESSIVE_MOOD", "DEPRESSIVE_MOOD", ifelse(newdata$dx == "SUD", "SUD","OTHER"))))

View(newdata) #make sure “View" starts with a capital V

#########################################################

#selecting variables to be analyzed

newdata = subset(newdata, select = c(readmission, sex, days, clos, dx, ddx, suicide, agegroup, remarks, work, discharge))

##########################################################

#SKIM DATA install.packages("skimr") library(skimr) skim(newdata)

str(newdata)

##########################################################

#CONVERT TO FACTORS newdata$sex=as.factor(newdata$sex) newdata$clos=as.factor(newdata$clos) newdata$suicide=as.factor(newdata$suicide) newdata$remarks=as.factor(newdata$remarks) newdata$dx=as.factor(newdata$dx) newdata$ddx=as.factor(newdata$ddx)

newdata$readmission=as.factor(newdata$readmission) newdata$agegroup=as.factor(newdata$agegroup)

newdata$work=as.factor(newdata$work)

newdata$discharge=as.factor(newdata$discharge)

str(newdata)

########################################################

#CHECK LEVELS OF THE VARIABLES

#this will also give the reference group/denominator levels(df$var)

#A RELEVELLING which gives a new reference group df$var=relevel(df$var, ref="level")

newdata$clos=relevel(newdata$clos, ref="SHORT")

#check to see if it has worked

table(newdata$clos)

#A RELEVELLING which gives a new reference group newdata$work=relevel(newdata$work, ref="UNEMPLOYED")

#check to see if it has worked

table(newdata$work)

#this will give levels with counts

#A RELEVELLING which gives a new reference group

newdata$remarks=relevel(newdata$remarks, ref="DISCHARGED")

#check to see if it has worked

table(newdata$remarks)

#A RELEVELLING which gives a new reference group

newdata$discharge=relevel(newdata$discharge, ref="OTHER")

#check to see if it has worked

table(newdata$discharge)

#A RELEVELLING which gives a new reference group

newdata$dx=relevel(newdata$dx, ref="PSYCHOTIC")

#check to see if it has worked

table(newdata$dx)

#A RELEVELLING which gives a new reference group

newdata$sex=relevel(newdata$sex, ref="M")

#check to see if it has worked

table(newdata$sex)

skim(newdata)

str(newdata)

###########################################################

#TIME FOR GTSUMMARY!!

install.packages("tidyverse") install.packages("gtsummary")

library(tidyverse)

library(gtsummary)

newdata %>% tbl_summary(

by = clos,

type = all_continuous() ~ "continuous2", statistic = all_continuous() ~ c(

"{mean} ({sd})",

"{median} ({p25}, {p75})",

"{min}, {max}"

),

missing = "ifany", missing_text = "Data missing"

)%>%

add_p(pvalue_fun = ~ style_pvalue(.x, digits = 2)) %>% add_overall() %>%

add_n() %>%

modify_header(label ~ "**Variable**") %>%

modify_spanning_header(c("stat_1", "stat_2") ~ "**LENGTH OF STAY**") %>% modify_footnote(

all_stat_cols() ~ "Median (IQR) or Frequency (%)"

) %>%

modify_caption("**Patient Characteristics**") %>% bold_labels()

#UVlogistic regression expressed as a single table

newdata %>%

select(clos, agegroup, sex, work, ddx, dx, suicide, readmission, discharge, remarks

) %>% tbl_uvregression(

method = glm, y = clos,

method.args = list(family = binomial), exponentiate = TRUE,

pvalue_fun = ~ style_pvalue(.x, digits = 2)

) %>%

modify_header(label = "**Variable**")%>% modify_caption("**univariate regression analysis**")%>%

add_global_p() %>% # add global p-value

add_nevent() %>% # add number of events of the outcome

add_q() %>% # adjusts global p-values for multiple testing

bold_p() %>% # bold p-values under a given threshold (default 0.05)

bold_p(t = 0.10, q = TRUE) %>% # now bold q-values under the threshold of 0.10 bold_labels()

#> add_q: Adjusting p-values with

#> `stats::p.adjust(x$table_body$p.value, method = "fdr")`

##################################################################

#collinearity check in logistic regression

logit_model <- glm(clos ~ dx+ ddx+ readmission+ agegroup+ sex+

work+ suicide+ discharge, data = newdata, family = binomial) install.packages("car")

car::vif(logit_model)

##################################################################

#multivariable logistic regression using gtsummary package

MODEL= glm(clos ~ dx + work + ddx + agegroup + sex + discharge + readmission + suicide, data = newdata, family = binomial)

tbl_regression(MODEL, exponentiate = TRUE)

##################################################################

#reanalyzing the model w/o gtsummary for comparison

MODEL= glm(clos ~ dx + work + ddx + agegroup + sex + discharge + readmission + suicide, data = newdata, family = binomial)

summary(MODEL)

round( cbind( ORs=exp(coef(MODEL)), exp(confint(MODEL))), digits=2)

##################################################################

#Report on model

install.packages("report") install.packages("performance")

library(report)

report(MODEL)%>% summary()

#Check model performance

library(performance) model_performance(MODEL)

###################################################################

#Goodness of fit test for a logistic regression

install.packages("rcompanion")

library(rcompanion)

#Fit the logistic regression model

MODEL <- glm(clos ~ dx + work + ddx + agegroup + sex + discharge + readmission + suicide, data = newdata, family = binomial)

nagelkerke(MODEL) #will return McFadden, Cox and Snell, Nagelkerke, Likelihood Ratio Test results ####################################################################

#multivariable logistic regression examining factors associated with hx of previous admission using gtsummary package

library(tidyverse)

library(gtsummary)

MODEL= glm(readmission ~ dx + work + ddx + agegroup + sex + discharge + clos + suicide, data = newdata, family = binomial)

tbl_regression(MODEL, exponentiate = TRUE)

######################################################################

#getting number of missing observations

# Assuming your dataframe is named 'dataset'

# Calculate the percentage of missing values for the entire dataset

#method 1

colSums(is.na(newdata))/nrow(newdata)*100 total_missing <- mean(is.na(newdata)) * 100

print(total_missing)

#method 2

total_missing_percentage <- mean(is.na(newdata)) * 100

# Display the result

cat("Percentage of missing values in the entire dataset:", round(total_missing_percentage, 2), "%\n") #####################################################################

#This code disaggregates continuous data, makes continuous data the outcome.

#can change statistic to mean.

#statistic is tricky, for some reason I cannot combine "{median} ({p25}, {p75})"

#and "{mean} ({sd})" library(gtsummary) tbl_continuous( data= newdata, variable = days,

include = c(dx, ddx, readmission, remarks, agegroup, sex, suicide, work), digits = everything() ~ 1,

by = NULL,

statistic = ~ "{median} ({p25}, {p75})", label = NULL ) %>%

modify_caption("**Patient Characteristics**")%>% modify_header(label ~ "**Variable**")

tbl_continuous( data= newdata, variable = days,

include = c(dx, ddx, readmission, remarks, agegroup, sex, suicide, work), digits = everything() ~ 1,

by = NULL,

statistic = ~ "{mean} ({sd})", label = NULL ) %>%

modify_caption("**Patient Characteristics**")%>% modify_header(label ~ "**Variable**")

###############################

#finding mean, median and range

mean(df$var, na.rm = TRUE) mean(newdata$days, na.rm = TRUE) median(newdata$days, na.rm = TRUE) range(newdata$days, na.rm = TRUE) ######################################

#Report R version and packages used, including citations library(report)

report(sessionInfo())

report_packages()

#############END###################

Analyses were conducted using the R Statistical language (version 4.3.2; R Core Team, 2023) on macOS Sonoma 14.1.2, using the packages below:

1. lubridate (version 1.9.3; Grolemund G, Wickham H, 2011)
2. performance (version 0.10.8; Lüdecke D et al., 2021)
3. report (version 0.5.8; Makowski D et al., 2023)
4. rcompanion (version 2.4.34; Mangiafico SS, 2023)
5. tibble (version 3.2.1; Müller K, Wickham H, 2023)
6. R (version 4.3.2; R Core Team, 2023)
7. gtsummary (version 1.7.2; Sjoberg D et al., 2021)
8. skimr (version 2.1.5; Waring E et al., 2022)
9. ggplot2 (version 3.4.4; Wickham H, 2016)
10. forcats (version 1.0.0; Wickham H, 2023)
11. stringr (version 1.5.1; Wickham H, 2023)
12. tidyverse (version 2.0.0; Wickham H et al., 2019)
13. readxl (version 1.4.3; Wickham H, Bryan J, 2023)
14. dplyr (version 1.1.4; Wickham H et al., 2023)
15. purrr (version 1.0.2; Wickham H, Henry L, 2023)
16. readr (version 2.1.4; Wickham H et al., 2023)
17. tidyr (version 1.3.0; Wickham H et al., 2023)

Package References

----------

1. Grolemund G, Wickham H (2011). “Dates and Times Made Easy with lubridate.” _Journal of Statistical Software_, *40*(3),1-25. <https://www.jstatso .org/v40/i03/>.

1. Lüdecke D, Ben-Shachar M, Patil I, Waggoner P, Makowski D (2021). “performance: An R Package for Assessment, Comparison and Testing of Statistical Models.” _Journal of Open Source So ware_, *6*(60), 3139. doi:10.21105/joss.03139 <https://doi.org/10.21105/joss.03139>.

1. Makowski D, Lüdecke D, Patil I, Thériault R, Ben-Shachar M, Wiernik B (2023). “Automated Results Reporting as a Practical Tool to Improve Reproducibility and Methodological Best Practices Adoption.” _CRAN_. <https://easystats.github.io/report/>.

1. Mangiafico SS (2023). _rcompanion: Functions to Support Extension Education Program Evaluation_. Rutgers Cooperative Extension, New Brunswick, New Jersey. version 2.4.34, <https://CRAN.Rproject.org/package=rcompanion/>.

1. Müller K, Wickham H (2023). _tibble: Simple Data Frames_. R package version 3.2.1, <https://CRAN.R-project.org/package=tibble>.

1. R Core Team (2023). _R: A Language and Environment for Statistical Computing_. R Foundation for Statistical Computing, Vienna, Austria. <https://www.R-project.org/>.

1. Sjoberg D, Whiting K, Curry M, Lavery J, Larmarange J (2021). “Reproducible Summary Tables with the gtsummary Package.” _The R Journal_, *13*, 570-580. doi:10.32614/RJ-2021-053 <https://doi.org/10.32614/RJ-2021-053>, <https://doi.org/10.32614/RJ-2021-053>.

1. Waring E, Quinn M, McNamara A, Arino de la Rubia E, Zhu H, Ellis S (2022). _skimr: Compact and

Flexible Summaries of Data_. R package version 2.1.5, <https://CRAN.R-project.org/package=skimr>.

1. Wickham H (2016). _ggplot2: Elegant Graphics for Data Analysis_. Springer-Verlag New York. ISBN 9783-319-24277-4, <https://ggplot2.tidyverse.org>.

1. Wickham H (2023). _forcats: Tools for Working with Categorical Variables (Factors)_. R package version 1.0.0, <https://CRAN.R-project.org/package=forcats>.

1. Wickham H (2023). _stringr: Simple, Consistent Wrappers for Common String Operations_. R package version 1.5.1, <https://CRAN.R-project.org/package=stringr>.

1. Wickham H, Averick M, Bryan J, Chang W, McGowan LD, François R, Grolemund G, Hayes A, Henry L,

Hester J, Kuhn M, Pedersen TL, Miller E, Bache SM, Müller K, Ooms J, Robinson D, Seidel DP, Spinu V,

Takahashi K, Vaughan D, Wilke C, Woo K, Yutani H (2019). “Welcome to the tidyverse.” _Journal of Open Source So ware_, *4*(43), 1686. <https://doi.org/10.21105/joss.01686>.

1. Wickham H, Bryan J (2023). _readxl: Read Excel Files_. R package version 1.4.3, <https://CRAN.R-project.org/package=readxl>.

1. Wickham H, François R, Henry L, Müller K, Vaughan D (2023). _dplyr: A Grammar of Data Manipulation_. R package version 1.1.4, <https://CRAN.R-project.org/package=dplyr>.

1. Wickham H, Henry L (2023). _purrr: Functional Programming Tools_. R package version 1.0.2, <https://CRAN.R-project.org/package=purrr>.

1. Wickham H, Hester J, Bryan J (2023). _readr: Read Rectangular Text Data_. R package version 2.1.4, <https://CRAN.R-project.org/package=readr>.

1. Wickham H, Vaughan D, Girlich M (2023). _tidyr: Tidy Messy Data_. R package version 1.3.0, <https://CRAN.R-project.org/package=tidyr>.
